# Evaluation of the Quality of Life in Moroccan Patients Diagnosed with Hypoparathyroidism

**DOI:** 10.1155/2024/7337895

**Published:** 2024-04-16

**Authors:** Mohammed-Amine Essafi, Samira Handa, Hayat Aynaou, Houda Salhi

**Affiliations:** ^1^Department of Endocrinology, Diabetology, Metabolic Diseases and Nutrition, Hassan II University Hospital Center, Fes, Morocco; ^2^Laboratory of Epidemiology, Research in Health Sciences, Fez, Morocco; ^3^Faculty of Medicine and Pharmacy, USMBA, Fez, Morocco

## Abstract

**Objective:**

Hypoparathyroidism (HPt) is a rare endocrine disorder often of postsurgical origin, resulting in hypocalcaemia. Several complications have been described including impairment of quality of life (QoL). Our study aims to evaluate the effect of hypoparathyroidism on the QoL of patients diagnosed with HPt.

**Methods:**

A cross-sectional study was conducted in the Department of Endocrinology, Diabetology, Metabolic Diseases and Nutrition of the Hassan II University Hospital of Fez. We included in our study all patients followed for chronic HPt. Well-being was assessed using the WHO 5 index, and QoL was assessed by the SF-36 questionnaire in its validated Arabic version. Data were entered into Excel and analysed using SPSS 26. Multiple linear regression was utilized to ascertain the variables linked to the QoL in individuals diagnosed with HPt.

**Results:**

A total of 143 patients with HPt were included in the study, 86.7% of whom were female. The mean age of the patients was 44.6 ± 17.3 years. 89.9% were of postsurgical etiology. The assessment of well-being by the WHO 5 index showed a low score (<50), meaning poor well-being in 44.8%. Regarding the QoL, the assessment showed low scores in the areas of general health (41.7), limitations due to physical condition (40.5), vitality (41.4), and limitations due to psychological condition (42.6). The multiple linear regression model revealed a noteworthy association between low SF-36 score and advanced age (*β* = −5.91; *p* < 0.001), surgical etiology (*β* = 8.71; *p* < 0.001), low education level (*β* = −10.1; *p* < 0.001), and poor compliance with medication (*β* = −11.3; *p* < 0.001). However, the relationship between impaired QoL and achievement of normocalcemia was nonsignificant (*p*=0.69).

**Conclusions:**

Our work objective is that patients with HPt have a reduced and multifactorial QoL. Despite normocalcemia, it is hypothesized that parathyroid hormone directly influences QoL. These results could serve as a basis for future research.

## 1. Introduction

Hypoparathyroidism (HPt) is a rare endocrine disorder responsible for hypocalcemia and hyperphosphatemia due to insufficient levels of parathyroid hormone (PTH) [1]. The most common causes of HPt are postsurgical complications, genetic or autoimmune diseases [2]. HPt often arises from thyroid surgery, primarily due to inadvertent damage to the parathyroid glands [3, 4].

The prevalence of HPt in the general population is not adequately documented. A Danish study found a prevalence of 24 cases of HPt per 100,000 population, with 22 postsurgical cases [5]. The reported incidence of chronic postsurgical HPt is highly variable, ranging from 0.3% to 17% [5, 6].

In the realm of endocrine disorders, chronic HPt stands out as the sole condition where conventional treatment does not involve hormone replacement. Instead, it necessitates lifelong supplementation with calcium and active vitamin D analogs [7, 8], coupled with regular monitoring. Nonetheless, the administration of calcium and vitamin D supplements does not fully reinstate the physiological balance of calcium and phosphorus [9].

Numerous short- and long-term complications have been documented. Büttner et al. [9] underscored a noteworthy supplementary aspect of HPt, specifically the diminished quality of life (QoL) [9]. Assessment of QoL in patients with HPt can help practitioners with optimal management and thus improve communication with patients to increase satisfaction and adherence to care.

As far as our awareness extends, no prior study has examined the influence of chronic HPt on the QoL among Moroccan patients.

Consequently, our study aims to evaluate the QoL and explore its influencing factors in Moroccan patients diagnosed with HPt.

## 2. Methods

### 2.1. Study Design

A cross-sectional study was conducted at the Department of Endocrinology, Diabetology, Metabolic Diseases and Nutrition of the Hassan II University Hospital of Fez, over the period between January 2021 and January 2022.

The Hassan II University Hospital of Fez is a regional reference center, and all patients with chronic HPt are treated and followed up in our department.

### 2.2. Population

#### 2.2.1. Inclusion Criteria

Our study encompassed all patients aged 18 years followed for chronic HPt in our database of patients hospitalized in the department from 2010. HPt was characterized by hypocalcemia and low or inadequate serum PTH.

Confirmation of chronic HPt was established if the condition endured for a duration of ≥6 months and/or if the patient necessitated ongoing treatment with active vitamin D to sustain normocalcemia.

**Figure 1:**
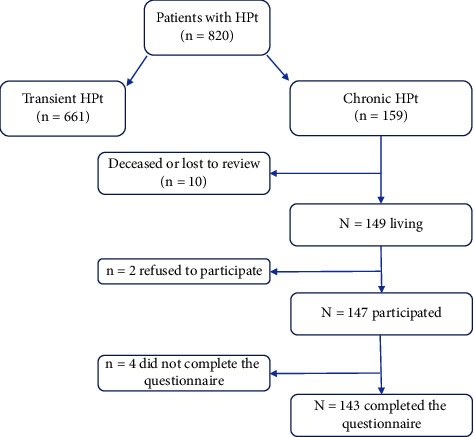
Study flow-chart.

The patients were contacted, and after elimination of the exclusion criteria, they were called during the period from January 2021 to January 2022 for a face-to-face interview (Figure 1).

#### 2.2.2. Exclusion Criteria

We excluded patients monitored for temporary hypoparathyroidism (those not meeting the criteria for chronic HPt), those with psychiatric conditions, difficulties in comprehension or communication, incomplete medical records, and those who declined participation.

### 2.3. Data Collection

Information was gathered from the medical records of patients diagnosed with chronic HPt under the care of the Department of Endocrinology, Diabetology, Metabolic Diseases, and Nutrition at Hassan II University Hospital in Fez.

Well-being was assessed using the WHO 5 index, and QoL was assessed by the SF-36 questionnaire in its validated Arabic version. The collected data were recorded on an anonymous datasheet and subsequently incorporated into a computerized database.

The data used to support the findings of this study are available from the corresponding author upon request.

#### 2.3.1. Sociodemographic Variables

Age, sex, education level, and smoking status are the sociodemographic variables.

#### 2.3.2. Clinical and Anthropometric Variables

Comorbidities such as renal disease, neuropsychiatric conditions, musculoskeletal disorders, and cardiovascular diseases were checked. Additionally, we measured blood pressure, body mass index (BMI), and waist circumference.

Physical symptoms, cognitive, and emotional were assessed using a standard questionnaire based on patient-reported complaints.

#### 2.3.3. Paraclinical Variables

The results of X-rays (renal ultrasound, X-rays of limbs if necessary) and biological tests (calcium, albumin, PTH, phosphorus, vitamin D, magnesium, and others when necessary) were checked.

Normocalcemia is defined as a blood calcium level in the low normal range of the laboratory where the patient performed the test without causing hypercalciuria. Normocalcemia checked over the last 3 visits.

In general, the biological assessment brought during the consultation is recent, within the last week.

#### 2.3.4. Questionnaires Used

Two questionnaires were used.

*(1) Well-Being Was Assessed Using the WHO 5 Index*. Five questions inquire about the patient's feelings over the preceding two weeks [10]. Six response modalities on a frequency scale are possible: all the time (5), most of the time (4), more than half of the time (3), less than half of the time (2), occasionally (1), and never (0). An overall score is obtained by adding the responses to the five items, then multiplying this result by 4. The score ranges from 0 to 100, with a higher score indicating a better state of well-being. A threshold of less than 50 is commonly employed as a screening criterion for identifying individuals at risk of depression.

*(2) QoL Was Assessed by the SF-36 Questionnaire in Its Arabic Version*. The SF-36 questionnaire consists of 36 inquiries that address 8 domains related to both physical and mental health: physical functioning, role limitations due to physical health problems [11], physical pain, general health, vitality, social functioning, role limitations due to emotional health problems, and mental health. Scores on each of the subscales range from 0 to 100, where elevated scores signify enhanced physical functioning and psychological well-being.

#### 2.3.5. Therapeutic Data

Drugs and doses of conventional treatment: supplementation with calcium and active vitamin D analogues (Alfacalcidol).

Therapeutic adherence was defined as acceptance of and compliance with treatment as prescribed.

### 2.4. Statistical Analysis

Descriptive statistics were employed to depict sociodemographic information, clinical details, and QoL scores. Mean values and standard deviations represented quantitative variables, while percentages characterized qualitative variables.

Univariate analysis was utilized to identify factors linked to QoL, followed by multivariate analysis employing multiple linear regressions to ascertain factors associated with the scores. The significance threshold was set at a *p* value <0.05. Statistical analyses were conducted using SPSS 26.0 software.

### 2.5. Ethics Statement

All participants were treated with respect for anonymity and confidentiality. Informed consent was secured.

## 3. Results

### 3.1. Population Characteristics

159 patients were with chronic HPt, 149 were alive, and 143 patients completed the SF-36 questionnaire, resulting in a participation rate of 89.9% (Figure 1).

The characteristics of the 143 patients are shown in Table 1. The mean age of the patients was 44.6 ± 17.3 years. There was a clear female predominance (86.7%).

### 3.2. Assessment of Well-Being by the WHO 5 Index

**Figure 2:**
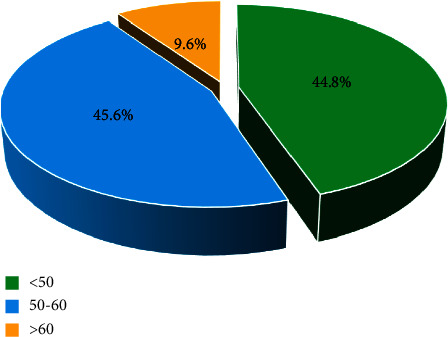
Assessment of well-being by the WHO 5 index.

The evaluation of well-being using the WHO 5 index revealed that 44.8% had a score below 50 [10], indicating a low level of well-being (Figure 2).

A score below 50, indicating poor well-being, exhibited a significant correlation with advanced age (>60 years, *p* < 0.001) and the postsurgical etiology of HPt (*p*=0.01).

### 3.3. SF-36 (Short Form 36) QoL Assessment

The symptoms reported by patients with HPt are summarized in Table 2 [11].

**Figure 3:**
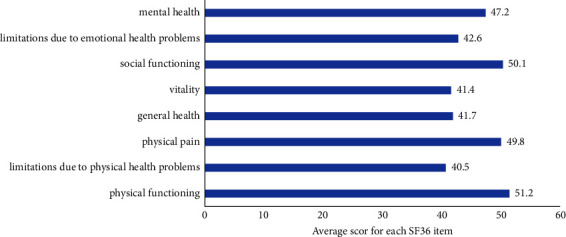
QoL in HPt patients according to the SF-36 score (scores on each of the subscales range from 0 to 100, where elevated scores signify enhanced physical functioning and psychological well-being).

The average total score of the SF-36 was 45.5 ± 9.7. The assessment revealed diminished scores in categories such as general health (41.7), physical condition-related limitations (40.5), vitality (41.4), and psychological condition-related limitations (42.6) (Figure 3).

### 3.4. Factors Associated with QoL

The outcomes of the univariate analysis demonstrated a noteworthy correlation between diminished QoL (indicated by a lower SF-36 total score) and advanced age (>60 years, *p* < 0.001), postsurgical etiology (*p*=0.004), inadequate therapy adherence (*p* < 0.031), as well as lower doses of calcium (*p*=0.013) and active vitamin D supplementation (*p*=0.012). However, the association between QoL and the attainment of normocalcemia was not statistically significant (*p*=0.69) (Table 3).

Multivariate analysis by multiple linear regression revealed that the items significantly associated with lower HPt-related quality of life were advanced age (*β* = −5.91; *p* < 0.001) and surgical origin (*β* = 8.71; *p* < 0.001), whereas medication adherence and advanced education were significantly associated with better QoL (*β* = −11.3 and −10.1, respectively, *p* < 0.001), after adjustment for all variables.

## 4. Discussion

Our study found impaired QoL in patients with chronic HPt, with reduced well-being.

Research on the QoL of patients with HPt is relatively recent. Various publications, including a recent systematic review [9], have highlighted compromised QoL in patients with HPt undergoing “conventional treatment” with calcium and active vitamin D.

In these studies, the QoL outcomes for patients with HPt are assessed by comparing them either to suitable control groups or to the general population.

The first study examining the QoL in HPt originates from Germany. In this study, 25 women with postsurgical HPt were compared to 25 women who had undergone thyroidectomy without developing HPt. The findings indicated that women with postsurgical hypoparathyroidism reported a lower sense of well-being compared to the control group [12].

In our study, patients with chronic HPt exhibited diminished scores in all domains of the SF-36 questionnaire, indicating a reduced quality of life. Our findings align with other studies utilizing the same SF-36 questionnaire [13, 14], including Cusano et al. [13], who studied 54 patients with postsurgical and autoimmune HPt. They observed lower QoL compared to the baseline population. Conversely, some studies have demonstrated significant impairment in only specific areas [9, 15–18].

What is particularly interesting in our study is that among the factors associated with reduced QoL, well-being was the postsurgical etiology. This can be explained by concomitant hypothyroidism, as hypothyroidism has already been shown to affect quality of life despite being on L-thyroxine treatment [19]. Thus, our study implies that a combination of HPt and hypothyroidism has a greater impact on QoL than HPt itself, in line with comparable studies [12, 15].

In our findings, an association was observed between advanced age and lower quality of life (indicated by a low SF-36 score), a correlation that aligns with previous study results [9, 15]. Additionally, a higher level of education was identified as having a positive influence on the QoL for individuals with HPt. This observation is in line with findings from other studies [13, 17]. The reasoning behind this may be that individuals with a higher level of education tend to exhibit better compliance and self-management of the disease.

In our study, the lack of a significant association between impaired quality of life and the achievement of normocalcemia (*p*=0.69) is consistent with research indicating that, despite calcium and vitamin D supplementation, many patients continue to experience physical, mental, or emotional symptoms [20]. This suggests that parathyroid hormone (PTH) deficiency itself may directly influence the compromised quality of life. PTH receptors have been identified in various regions of the central nervous system [21, 22], and PTH deficiency could impact different aspects of QoL. Investigations into PTH replacement therapy [13–24] have shown promising outcomes. Further studies are necessary to explore the effects of PTH treatment.

A notable strength of our study is the inclusion of an unselected group, encompassing all patients diagnosed with hypoparathyroidism (HPt). This approach renders the sample representative of all causes of HPt, and the study benefits from a relatively robust sample size. Additionally, comprehensive medical and surgical records were accessible for the current research.

Certain limitations should be acknowledged, particularly the face-to-face questionnaires. This subjective nature of the data introduces the potential for influence or bias.

## 5. Conclusion

Our study aimed to assess the quality of life in patients with hypoparathyroidism (HPt) undergoing standard treatment, revealing impairment despite stable calcium and vitamin D levels. Several factors, including advanced age and a postsurgical etiology, were associated with reduced quality of life, while a higher education level correlated with better quality of life.

It is hypothesized that parathyroid hormone deficiency directly impacts the quality of life, prompting interest in recommending recombinant PTH for HPt management.

These findings could serve as a foundation for further research into the disease burden, encompassing a study of both the direct and indirect costs associated with the condition.

## Figures and Tables

**Table 1:** Descriptive data for patients with chronic HPt.

Characteristics	*N* = 143
Age	44.6 ± 17.3 years
Education level: illiterate	68 (47.5%)
Education level: primary	47 (32.8%)
Education level: university	28 (19.7%)
Etiology: postsurgical HPt, *n* (%)	128 (89.5%)
Etiology: nonsurgical HPt, *n* (%)	15 (10.5%)
BMI	24.6 ± 5.23 kg/m^2^
WC	92.3 ± 7.13 cm
TSH	2.1 ± 1.4 mIU/L (0.5–5)
Average dose of calcium/day	1720.87 ± 470.23 mg

HPt: hypoparathyroidism; BMI: body mass index; WC: waist circumference; TSH, thyroid stimulating hormone.

**Table 2:** Symptoms reported by patients with HPt.

Physics (%)	Cognitive (%)	Emotional (%)
Asthenia 79	Memory problems 84	Anxiety 97
Pain 82	Concentration disorder 96	Mood disorder 93
Paraesthesia 92		

**Table 3:** QoL of patients with chronic HPt.

Variables	Number (%) (*N* = 143)	Total score SF-36 (mean ± SD)	*p* value
Age >60 years	109 (76.3%)	38.08 ± 13.62	**<0.001**
Age ≤60 years	34 (23.7%)	49.67 ± 14.7	

Gender: male	19 (13.8%)	42.08 ± 13.7	0.76
Gender: female	124 (86.7%)	43.23 ± 11.12	

Marital status: single	24 (16.8%)	45.12 ± 14.2	0.45
Marital status: married	119 (83.2%)	43.34 ± 10.6	

Postsurgical etiology	128 (89.5%)	41.32 ± 5.72	**0.004**
Nonsurgical etiology	15 (10.5%)	50.13 ± 7.12	

Dose of calcium/day ≥2 g/day	109 (76.2%)	49.1 ± 3.3	**0.013**
Dose of calcium/day <2 g/day	34 (23.8%)	43.2 ± 8.6	

Dose of alfacalcidol/day ≥1.5 *μ*g/day	111 (77.6%)	47.1 ± 3.15	**0.012**
Dose of alfacalcidol/day <1.5 *μ*g/day	32 (22.4%)	40.4 ± 6.72	

The duration of disease: ≤10 years	85 (59.4%)	46.2 ± 8.3	0.82
The duration of disease: >10 years	58 (40.6%)	44.7 ± 7.9	

Normocalcemia: yes	101 (70.6%)	47.7 ± 9.72	0.69
Normocalcemia: no	42 (29.4%)	46.5 ± 10.15	

Therapeutic compliance: yes	99 (69.2%)	49.6 ± 5.72	**<0.031**
Therapeutic compliance: no	44 (30.8%)	41.3 ± 4.15	

HPt: hypoparathyroidism. Bold values represent significant p values.

## Data Availability

The data used to support the findings of this study are available from the corresponding author upon request.
